# A biophysical model of cerebellar molecular layer interneuron

**DOI:** 10.1186/1471-2202-14-S1-P96

**Published:** 2013-07-08

**Authors:** Shyam Kumar Sudhakar, Erik De Schutter

**Affiliations:** 1 Department of Theoretical Neurobiology and Neuroengineering, University of Antwerp, Wilrijk, 2610, Belgium; 2 Okinawa Institute of Science and Technology, Okinawa, Japan

## 

The molecular layer of the cerebellum is characterized by small interneurons (basket and stellate cells). They exert a strong inhibition on their postsynaptic Purkinje cell targets [1]. They are known to be present in large numbers in the molecular layer. They outnumber the Purkinje cells by a factor of 10 [2]. Gap junctions between them characterize the interneurons of the cerebellar cortex. The interneurons are also known to inhibit each other. The extensive connectivity network between the interneurons and with that of the Purkinje cells, make the interneurons of the molecular layer a vital component in determining the output of the Purkinje cells.

We constructed a biophysical model of molecular layer interneuron of the cerebellar cortex in order to study them more extensively at network level. The model was constructed using NEURON 7.2 simulation environment. The model is characterized by the presence of sodium channel, a non-inactivating potassium channel, Kv4.3 potassium channel, a low threshold T-type calcium channel and a hyperpolarization activated cation current. The model's input resistance and input capacitance were tuned to that of the experimental data [3,4]. The model has an input capacitance value of 9.5 pF and an input resistance of 670-680 MΩ. We also compared the spike output of the model with that of the experimental data [6]. The model closely follows the experimental data for all values of injected current. We also tested the model for initial spike latency since stellate cells express members of Kv4 potassium channel family [5]. The model displayed a non-monotonic relationship between first spike delay and membrane potential with longer delays to first spike recorded for intermediate values of membrane potential similar to that of experimental data [6]. However, the model peak spike amplitude is too high, an area of future improvement.

This biophysical implementation of molecular layer interneuron will be used in a cerebellar cortex network model to investigate how their activity impacts target Purkinje neurons and how this is modified by gap junctions.
